# Cultural Competence of Professionals Working With Unaccompanied Minors: Addressing Empathy by a Shared Narrative

**DOI:** 10.3389/fpsyt.2020.00528

**Published:** 2020-06-11

**Authors:** Rahmeth Radjack, Fatima Touhami, Laure Woestelandt, Sevan Minassian, Yoram Mouchenik, Jonathan Lachal, Marie Rose Moro

**Affiliations:** ^1^ AP-HP, Hôpital Cochin, Maison de Solenn, Paris, France; ^2^ Université Paris-Saclay, UVSQ, Inserm, CESP, Team DevPsy, Villejuif, France; ^3^ UTRPP, University Sorbonne Paris Nord, Paris, France; ^4^ University of Paris, Sorbonne Paris Cité, Paris, France

**Keywords:** unaccompanied migrant youth, cultural competence, transcultural approach, migration, trauma, social work

## Abstract

**Background:**

The number of migrant youth traveling without parents continues to rise in Europe and North America. Some of t hem leave their home countries on their own and find themselves in a new country, separated from their family and cut off from their cultural roots. Besides those who leave to study, work, and pursue a better life, others are escaping war-torn countries. They need adequate social, educational, and therapeutic spaces, where they can feel entitled to speak. Social workers often ask about how they can understand these young people better so that they can provide them with better care (cope with their trauma and suspicion, deal with the cultural distance between the adolescents and their social workers, etc).

**Aim:**

At Cochin Hospital in Paris, we led a participative action-research program to transmit cultural competence to social workers who provide care for these youth. The aim was to develop an approach to help these young migrants to share their representations about themselves and to train these social workers to encourage this sharing in a culturally sensitive manner.

**Methods:**

This study used a qualitative method that mixed narrative and transcultural approaches. Two researchers met each youth and social worker with an interpreter-cultural mediator three times (once a month) to assess changes in their relationships during the study. The youth were asked to bring three items of their choice, representing their past, present, and future. They could use their imagination and creativity. We also used the circle test described by Cottle for this purpose. We used a phenomenological approach to analyze the interviews.

**Results and Discussion:**

This study included 29 young people from 13 different countries and 29 social workers. A transcultural approach appears to be a useful framework for reactivating their identity construction process. It promotes the emergence of cultural representations and takes their experiences before, during, and after migration into account. We assisted them in developing their ability to produce a thorough narrative of their bicultural adolescences and simultaneously helped their social workers to develop their cultural competence.

**Conclusion:**

Together, a transcultural approach and methods stimulating the production of narrative are relevant ways to help children to describe their representations of themselves, especially those who have learned to protect themselves by remaining silent. This protocol could be useful for both preventive action and therapy for psychotrauma.

## Introduction

In France, unaccompanied immigrant minors are considered to be children unprotected by parents or other legal representatives. They often come from North Africa, the Indian subcontinent, or sub-Saharan Africa, after a perilous journey. Some have left their country and their family to study, work, and pursue a better life. Others are escaping war-torn countries. Some are seeking asylum, while others are not. They are separated from their families and cut off from their cultural roots. Most of them have faced traumatizing adverse events before, during, or after their journey. The mobilization of their psychological resources is thus essential to cope with the difficulties they confront, to ensure their survival, and to face the challenge of migration to a world they had idealized but did not know, one that disrupts their identity.

Despite the absence of clinical homogeneity, most of these teenagers have had to live alone through their identity construction period and have often experienced repeated trauma and multiple bereavements. The brutal and repetitive ruptures that they experience may generate mental illnesses, especially attachment or posttraumatic disorders. Studies report that 52% have posttraumatic disorders, 44% depressive syndromes, and 38% an anxiety disorder (1–4). For around a third of these adolescents, these disorders go on to become chronic (5), especially those with anxious or posttraumatic symptoms (6).

The only significant protective factors so far reported are optimal social work, school management, and long-distance family support (7–9). Accordingly, international publications about these young people (7, 10–13) underline the need to improve how professionals understand them and to provide better care to prevent these psychiatric disorders. Nonetheless, the professionals responsible for providing them with support often feel helpless as they require information to help them to understand and take action (14) to assist these unaccompanied migrant youth, whose issues differ greatly from those of teens traveling with their families. The social workers who work with them must cope with a variety of problems. In France, the Child Welfare Bureaus, responsible for providing services for them, are saturated because of the continuous increase in their number since the turn of the century.^1^ Lack of knowledge about the youth’s culture and lack of training about symptoms of trauma can lead to misinterpretation and misunderstanding. Furthermore, a degree of suspicion often interferes with the social work: these youths are regularly suspected of making up stories to obtain legal immigration status (15). The teens themselves may experience difficulties in trusting any adults, after the loss of their family or other various traumatic events, including being victimized by smugglers. The discontinuities in social work services do not promote either clarity about the social workers’ role or the quality of the services they provide. On arrival in France, these young people are first evaluated to verify that they are indeed minors and without family; they are then transferred successively to different sites (an emergency shelter, and then a more stable group home, or hotel or foster family). Finally, these youngsters confront a multiplicity of difficulties and paradoxes. Because their absence of adult protection prevails over their “foreignness,” they receive temporary legal protection and social services in France. At 18, however, they can be deported back to the chaotic environment that they fled, often at the risk of their lives. They are exposed to extremely discordant attitudes among the professionals who deal with them (due to the conflicts between immigration policy and child protection laws).

Professionals consider that overcoming these complex and specific problems requires transcultural skills, concepts defined by Domenig (16) and Althaus (17). The transcultural approach has developed over the past 20 years in France; it is based on the assumption that to understand and effectively care for migrants, it is necessary to take into account their cultural affiliations, their ways of thinking, but also their migration experience (12, 18, 19). Creativity is required for helping these young unaccompanied migrants whose families are far away (18, 20).

From a psychological perspective it is imperative to provide a setting where they feel welcomed and create spaces where they can express their needs and desires. Good communication is also essential in constructing a “working alliance” with them, especially at their arrival, when they are in the greatest need of information.

The *Maison des Adolescents* (French facilities providing integrated youth health care) (21) at Cochin Hospital (known as the *Maison de Solenn*) in Paris conducted a qualitative study^2^ from 2012 to 2016 of the construction of narratives, jointly by the young unaccompanied migrants whose life stories these are, and the social workers responsible for them. This research involved creating a situation where the youth and his or her social worker could meet and learn from each other and where we and the social workers could learn more about these youth and their journeys. It is part of a comprehensive interinstitutional project jointly conducted by clinical departments (*Maison de Solenn*-Hospital Cochin), a research group (INSERM U1178), and the Paris Child Welfare Bureau.

Our study had a twofold principal objective: 1) to develop an approach to help these youth describe their representations of themselves, as well as their journeys and their answers to transcultural questions from the narratives they provided, stimulated by objects they chose; and 2) simultaneously training the social workers who work with them to encourage this sharing in a culturally sensitive manner. Our aims were to identify how gain access to their perspectives, while taking into account their cultural representations, and to teach this skill to social workers. The secondary objective was to assess the youths’ experience of the treatment program we studied. We seek in particular to identity the aspects useful for the preventive or therapeutic management of psychological trauma in this population at risk.

## Materials and Methods

This is a qualitative study. The appropriate ethics committee (CEERB Paris North IRB00006477) approved this protocol in 2014.

During a *focus group* that took place during the exploratory portion of this study with our partners of the Child Welfare Bureau, care providers and social workers often expressed a sense that they were “missing something” in their encounters with these youth. These destabilizing professional experiences engendered countertransference reactions of helplessness and failure. We therefore constructed a research framework likely to facilitate the emergence of the teens’ discourse about accurate representations of themselves. We wanted to give them the chance to develop narratives that escape the preconceived representations associated with their status. The underlying idea was to initiate, when necessary, a process of change in social workers’ representations of the youth to whom they are providing services and support and to help the professional recognize when this process is necessary and how to trigger it. In the literature on transcultural competence, we rely on the concept of narrative empathy set forth by Domenig (16), who describes this as the ability to listen in a way that is kind and supportive.

For the young migrants, this method attempts to induce them to feel the continuity of their existence and to help them construct their identity while far away from the landmarks that helped construct it. The theoretical foundation comes from the psychoanalytic literature on psychological trauma and identity reconstruction (22–24); narrative identity (25–27); and transcultural psychotherapy (18). At stake is the adolescent’s resumption of the process of identity construction, which this self-narration is intended to facilitate.

### Participants and Sampling

The study took place in three different cities (Paris, Bobigny, and Lille) among the districts hosting the largest portion of unaccompanied minors. Recruitment took place through two gateways: either after information meetings in the institutions chosen with the assistance of the Child Welfare Bureau (facilities serving mainly unaccompanied minors in their group homes); or after a request for psychological care by social workers to our team of transcultural clinicians.^3^ Participation was proposed to social workers who asked how to get to know the young migrants better because they felt that that they did not understand this population, or were missing something, or that there were cultural misunderstandings or distrust. It was also suggested when some action for the youth appeared useful but long-term follow-up seemed unnecessary, so that a limited number of interviews would be most productive.

The inclusion criteria for the young migrants were: agreement to participate, with their signature attesting to informed consent after translation by the interpreter), aged 11 to 19 years, and the agreement of their institutions for the social workers’ participation. An information sheet about the study was provided to social workers and adolescents. This information was provided in the youth’s maternal language, with the help of an interpreter. Particular care was given to the information and explanation of the study, especially in the context of the differences in language and culture and the often traumatic context of migration. We used purposive sampling to include participants who were typical of the study population. Both the professionals and the youth people included signed consent forms. We excluded those teens for whom immediate psychiatric care (for an acute psychiatric illness) was more important than participation in research. The data have been anonymized.

#### Setting and Data Collection

A semistructured interview topic guide developed in the exploratory study was used to probe each youth’s cultural identity, journey, perception, and experience after migration. A focus group including several transcultural clinicians sought to identify the most relevant and simplest questions enabling the emergence of a discourse and showing an interest in the other’s culture. The semistructured interview was drafted and then reassessed by a group of 10 transcultural experts. The circle test was added after a preliminary study, as was a self-administered questionnaire for professionals about the difficulties they encountered and the strategies they used in transcultural situations, required of professionals who wanted to participate in this study.

The process involved three meetings led by two researchers (trained in transcultural clinical care and management of psychotrauma) in the presence of the referring social worker and an interpreter-mediator to coconstruct a life narrative. We relied on three tools to facilitate the narrative: objects, the circle test, and cultural mediators. Accordingly, the youth were never directly questioned about their journeys; they were free to choose the topics selected.

**Figure 1:**
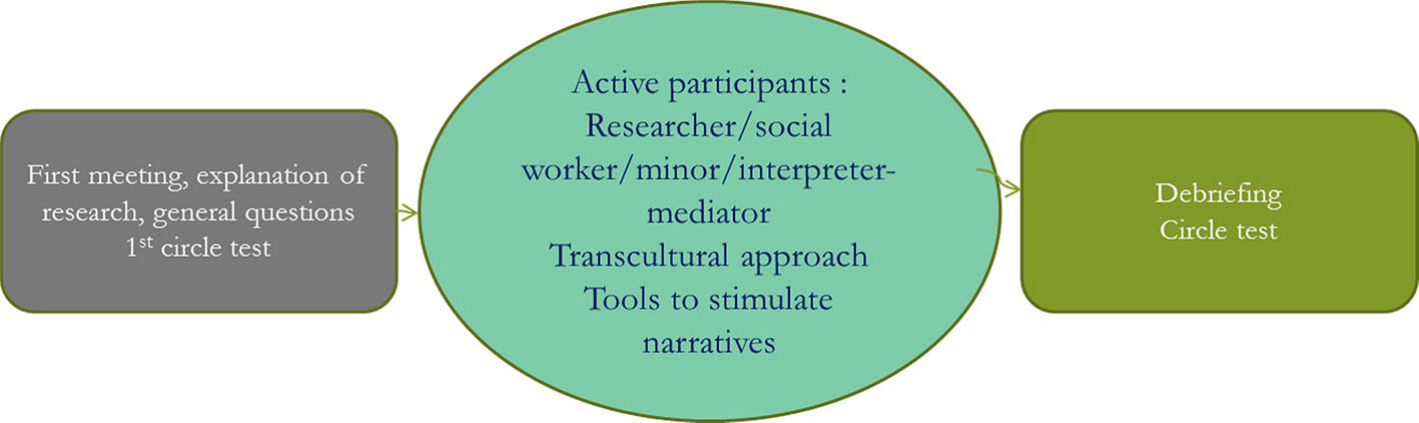
Three semistructured monthly interviews.

The three meetings took place, each a month apart (**Figure 1**). The interpreter-mediator, the minor, the referring social worker, and two researchers experienced in transcultural clinical care were present for all meetings. The first meeting began by asking the adolescent to take the Circle Test (**Table 1**), to test his or her spatial perception of time (28, 29). This test allowed the subjects to represent their perceptions of the past, present, and future, as well as the potential correlations between them.

**Table 1:** Instructions Circle test (28).

Instruction	*“Represent the past, present, and future in the form of circle”*
Guidelines	*“Draw three circles using the space of the white sheet, to represent the past, present, and future.* *Place them in the way that best represents what you feel about the relation between the past, present and future. You can use circles of different sizes”*
Legends	*“When you have finished, label each circle: the ones that represents the past, the present, and the future.”*
Comments	The adolescents are asked to comment on their circle tests

At the end of this first meeting, the teen was asked to bring three objects or thoughts (for example, some music, an image, a souvenir/memory, or a sentence) to the next meeting. The three objects were to be associated with the adolescent’s past, present, and future and were required to illustrate some aspects of their inner life, chosen randomly by context, mood, and the investment of the social worker. We were inspired by the floating objects used in a systemic approach (30, 31). This enabled the young participants to use their imagination and creativity. Methods stimulating the production of narrative are relevant ways to help children to describe their representations of themselves, especially those who have learned to protect themselves by remaining silent.

**Figure 2:**
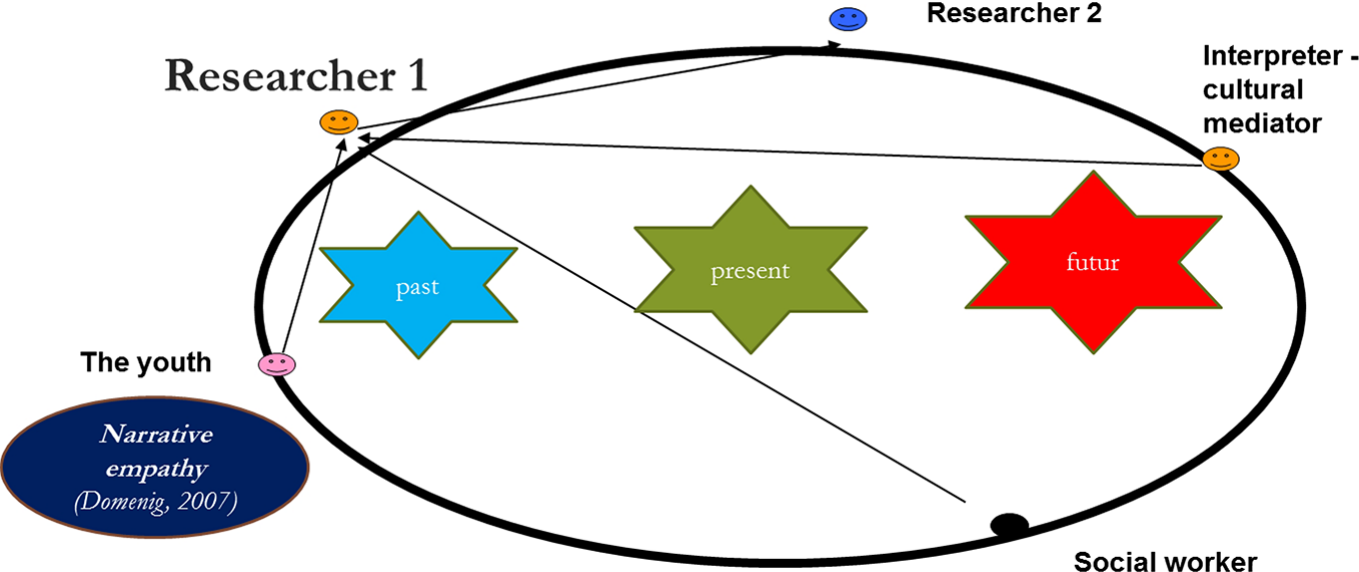
The research program.

The second meeting involved the exploration of the interaction between the adolescent and his or her social worker, in the presence of the interpreter-mediator.^4^ The objects provided the basis for the narrative. The researchers made transcultural propositions (18, 32, 33), while adopting narrative empathy, that is, listening attentively in a way that let the narrative move forward without being forced. Its emergence was facilitated by the active intervention of the mediator and the anthropological interpretation of the objects. The interpreter’s role is not solely to facilitate the progression of the narrative. The shared language and the interpreter’s knowledge of the world the youth comes from increase the possibilities of identification and promote the construction of the narrative by enabling an association between the youth’s past and present (**Figure 2**).

At the third meeting, conducted as a semistructured interview, each participant provided feedback about the overall experience. As in the first interview, the young participants were asked to take the “Circle Test” to see if any changes had occurred in their subjective experience of time from the beginning to the end of the study.

All interviews were audiorecorded and transcribed verbatim. All of the self-administered questionnaires were completed and used for our question-by-question analysis. The two sheets of the circle test (one the first month, and the other the third) were also collected for the analysis.

### Method of Analysis

The analysis—qualitative and thematic—is organized into two main themes: first, analysis of the life narratives, and second, the analysis of the changes in the narrative during the three interviews, as related to the mediators used (the circle test, the objects, and interpreters-mediators). A dual phenomenological and narrative approach is thus used (34–37).

The phenomenological approach is descriptive (35, 38). The analysis focused on the subjects’ narratives, the ways that they put their lives into a story to give it a meaning. The aim is thus to report the youths’ experiences holistically, preserving their richness and complexity. The analysis took place at several levels, as we attempted to demonstrate change and what drives it in the evolution of the discourse (content and form), to disengage its major themes, its context, and the individual, interpersonal, group, and societal connections.

The interview transcripts were analyzed thematically. They were read several times and then coded to identify the first themes. Initial codes were generated and then sorted into broader themes, with similar codes placed under the same theme. A theme was determined on the basis of its significance to the research question. Themes were then revised and refined to ensure codes within each theme were closely associated and that each theme was distinctive. Themes were then named. Procedural consistency was guaranteed by using a double-coding approach, with cross-checking between coders to ensure consistency. The youths’ names have been replaced by letters to protect their privacy. The analyses were performed simultaneously by one researcher who did not participate in the interviews and one who did. The researchers sought to distinguish the recurrent patterns, but they also integrated the emerging questions to take the convergences and divergences in the data into account. Divergences in assessment were resolved by a third researcher. The final stage is the production of a consistent ordered presentation of the themes that describe the participants’ experience (35).

The circle test was analyzed as described by Cottle (28): the sizes of the circles, the distance between them, their contents—all of that, juxtaposed to the narrative analysis of what was said about these circles and taking the transcultural context into account. We analyzed the changes of these circles between the first and the third interviews (39). The objects were analyzed from an anthropological perspective. The mediator assisted in the interpretation and in explaining the youth’s choice of object. The discourses around these objects were analyzed according to the content of the corresponding narrative, their poetic structures (themes, images, and metaphors), and the narrative itself (tone, rhythm, narrative coherence score) (40).

The final stage of the analysis involved writing a synthesis of the results. This synthesis includes several parts, each corresponding to a stage of the analysis. That is, the substantial quantity of data generated by this study requires the presentation of the specific results for each part of the analysis. Accordingly, the analyses restricted to a specific aspect have been the topic of academic work and/or publications by members of our research group, especially the analysis of the mediation by the objects (40) or by the *circle test* (39, 41, 42, 43), the difficulty of projection into the future (44), modifications in the social worker’s relationship with the youth between the three interviews (45, 46), the description of several life narratives (47–54), and the implementation of mental health care for distressed young migrants (55–57). Here, the research group proposes a cross-sectional analysis of the data useful for the preventive management of psychological trauma.

## Results and Analysis

The study included 29 young unaccompanied migrants and 29 social workers. Their sociodemographic profile varied by age, sex, and geographic origin, but was representative of the overall population of these youth. They were all aged 16 to 18 years, which is the most common age group for unaccompanied youth in France. The group includes only three girls, consistent with strong male sex distribution of this group in France. Their cultural origins are representative of the recent migration flows in France: sub-Saharan Africa, North Africa, and southern Asia. The difference between the age range included and the inclusion criteria is probably due to the fact that social workers find it more difficult to work with migrant youth approaching their 18^th^ birthday (because they receive care and support from the Bureau of Child Welfare only until then, except in exceptional situations). That is, the youth and the social worker must adjust/adapt to each other even more rapidly (language learning, administrative issues, entry into a rapid training program to be able to support him- or herself at the age of 18 years) and the challenge of support is still more complex. The social workers accordingly offered this study most often to these older youths.

Of the 29 subjects, 21 completed the three successive interviews. Two youths were unable to complete the second and third interviews due to severe psychiatric decompensation related to stressful contemporaneous events (their 18th birthdays and failure to obtain legal status). Others had to move away from Paris and leave their group home. Two ran away. Psychological distress was identified or mentioned before or during the research for 8 of 29 teenagers PTSD, depression, addictions, and somatic complaints. We then helped them to start treatment.

Their social workers also had a varied profile. Their professional experience working with unaccompanied minors ranged from 7 months to 4 years for the most experienced. They worked either in a group home or shelter or for an association or agency supporting youths housed in a hotel. Their decisions to suggest the youth’s participation in this study were based on their impressions of not adequately understanding their young client.

The analysis of the results shows two primary types of themes (**Table 2**): those that are part of the experience of being a young unaccompanied migrant, and those related to the value of this method in promoting changes in the youth and in the relationship between the youth and the social worker.

**Table 2:** Themes.

**The experience of being an unaccompanied minor**	- A suspended future - Religion: guarantee of continuity - Preserving one’s dignity - Difficulty of escaping a sense of loneliness
**Study impact**	- Taking into account the experiences before, during, and after migration - From “I” to “we” - A mediator that facilitates bridges between the cultural worlds

### The Experience of Being a Young Unaccompanied Migrant

#### A Suspended Future

For most of these adolescents, projection into the future was difficult. Their future felt suspended, subject to administrative and legal decisions, despite their hope of obtaining a residence permit, a diploma, or a job. It remained hypothetical for some, “*a hole, empty, there’s no future and that’s all”* for the young Y.

For three youths, especially, it was impossible to imagine an object associated with the future as long as their future remained uncertain. Nonetheless, the experience of the study and the creation of a narrative were described as positive by all of the participants who completed all three interviews. In the subgroup of youths who did not ask for psychological treatment, there was a clear positive evolution of the circle test between the first and third interviews; at the first interview, the present and future predominated, while in the second circle test, the largest circle is the future: *“an inflated future that is flying away and is filled it with important things, with a content (“women, children, parents, bank account, job*”).

Inversely, the circle tests did not change between the first and third interviews for those who needed psychological treatment, and the dominant time at the first interview was always the past. Nonetheless, the narrative around these circle tests evolved each time it was given: from a “transient present to a supportive present”, from a “past postponed to a past to be mastered,” from a “future unthought of” to a future that can be envisioned.” (“that of the future is bigger, I didn’t do it on purpose, it just came like that by itself”). We observed two psychiatric decompensations that were essentially brief psychotic episodes, although the first interviews did not include any notable elements that might have predicted them. These youth did not have any particular psychiatric history, but both turned 18 between the first and the second interviews and found their situations extremely precarious when they were no longer under the care of the Child Welfare Bureau (they had not obtained legal status). We can talk about wandering that can induce insanity and the impact of this sword of Damocles on their 18 birthday that seems to decide their future. We helped to organize hospitalization for these two youth, who came spontaneously to our facility (not for study interviews), having evidently recognized their welcome would be kind.

#### Religion: Guarantee of Continuity

Religion and more particularly the individual’s relationship to God and Islam was the theme found most frequently in these narratives. It was mentioned more specifically in discussing the past and the present. In the past, it was a link to the transmission of religious practices, by reading and, for some, learning verses of the Koran. It also testified to their membership in a tradition. At the present, it anchors them, especially as they continue, despite some difficulties, practices such as Ramadan in the host country. In most cases, God is a positive factor helping to alleviate an unknown future.

For example, Ad., from Senegal, brought a Koran for all three objects.

Describing the past, he explained, “in fact, since I was very young, I was born into it, the Koran; it’s what I learned first, well before I learned French or Arabic.” He had the best grades at his Koran school and was also the most adaptable of all his siblings. His father therefore chose him to go try his luck abroad.

For the present, he reported that the Koran represents a familiar object that he was able to find in France; it lets him remember who he is and encounter otherness with less anxiety. The Koran for him represents a reassuring and permanent object that helps him to face an uncertain future. He noted the importance of relying on thoughts or familiar places when one arrives in a land one does not know, with codes one has not learned. For the future, he said, he would use the Koran as a “guide for good behavior”, to give him his bearings in a new world still to be decoded.

Demonstrating his detailed knowledge of the Koran also enhanced his social worker’s impression of him. This simultaneously increased his own self-esteem, which is important in the creation of good bonds for these youths who often feel judged or undesirable in the host country and who thus feel their dignity attacked. Finally it allowed him to reconstruct with spontaneity a network to belong to, to feel less lonely, even to want again finally to belong to a group. At the last interview, the social worker was surprised at the changes in Ad., who was open and seemed to have more self-assurance after the acknowledgment of his worth. He was later able to join a theater group at his training center.

#### Preserving One’s Dignity

This theme was regularly associated in the narratives with recognition of the distress these youths have experienced. They allowed themselves to express the offense to their dignity although they had not succeeded in expressing it elsewhere. For example, M., a young Congolese from a well-to-do family describes discovering on his arrival in France how low on the social ladder he was now placed. His school showed its solidarity and found him lodgings. Nonetheless, none of the other students are aware of his situation.

Researcher: *He has to prove his identity, who he is. There is also this impossibility of speaking it and the weight of his need for success*.

Interpreter: *Especially in the region he’s from, men cannot show their feelings. From the age of 15 years, there, he is considered a man. A man, he must not cry, he must not show his feelings. So his honor is also at stake*.

Researcher: *He has a great deal of courage and dignity, and it is the case for many of these young minors who come from somewhere else, who suffer, but cannot tell their families their real situation in France*.

In another register, we were able to resolve a cultural misunderstanding for S., from India. He hadn’t dared to express the reasons he had refused a training program in horticulture, a program his social worker was quite proud of having obtained for him. In the presence of the interpreter-mediator and with the help of the researchers, who were able to get him to talk about his Sikh origins in relation to the objects he had brought (typical bracelets), he was able to explain that the crafts resulting in dirt stains on clothing were performed only by “castes inferior” to his. He thus felt humiliated and insisted on a training program in cooking, which his social worker had not understood.

#### Difficulties in Escaping a Feeling of Loneliness

Loneliness was another theme often found, despite the presence of people around them.

One adolescent quoted the Algerian proverb: “*One hand does not applaud*.” This experience was particularly intense in those youth who expressed psychological distress.

“*I always feel all alone, because at the difficult moments, there is no one here for me,*” F.

This deep feeling comes from the break with family, and sometimes from the difficulty of trusting: “*The community makes everyone think about itself, not help each other, so I can’t trust [anyone]*.” (Al., first interview)

“*By coming to Europe, I’ve left my place empty back there, I’ve lost my place.*” (Al.)

He thus faced an impasse: he had a vital need to be surrounded by people who care about him, but had not succeeded in meeting people he could count on in his current environment. This young Algerian was thus lost in his identity, somatizing his pain and using alcohol as an avoidance strategy.

Several teens stated that they do not want to worry their families, already in need. This aggravated their feelings of isolation, of loneliness. They have demonstrated their sensitivity to acknowledgment and recognition of their journey to uphold their dignity.

Interpreter: *“It’s El Dorado, that’s exactly it. You think that in arriving here, you just have to ask and you get. The problem with all that, it’s that the people who have immigrated here, who succeeded here, when they return to their country, they live like pashas, and that makes people think that in France, there’s a lot of money. So when people leave from there to come here, they think they’re going to El Dorado. Unfortunately here, there’s reality … All the minors I know, who I work with a lot, they often hide the problems from their family, because the family cannot imagine what happens here, and afterwards, they will worry too much. So the minor prefers to take it all on his own shoulders. But he believes that he has to succeed here and that he doesn’t have the right to fail.”*

Researcher: *“It’s a dual isolation.”*

Interpreter: *“And failure is forbidden.”*

The semistructured interviews made it possible to reconstruct the networks to which the youth belonged. The researchers made an effort to not think of these teens as isolated, by asking questions for example about the family, even though it is absent, and through the objects. The study aimed to make these adolescents want to belong once again to a group.

### Effects of This Research Program

#### Taking Experiences Before, During, and After Migration Into Account

The use of objects (**Supplementary Table 3**) and the circle test made it possible to avoid the cleavage of migration and, by systematically going back and forth between these three time periods, to help these adolescents recover the continuity between their past, present, and future. The items used to evoke narrative facilitated access to their life paths and their histories. They talked more spontaneously about aspects of their identity and their experience and expressed their emotions and feelings more easily than during the first interview, when they had brushed past or avoided these questions.

These youth had often passed through several countries and had had to adapt several times to different changes and risks.

“*For example I had 7 sets of identity papers, and there were two that were in Arabic and in Spanish.”*

The cleavage of migration that follows the trauma of exile often makes it difficult to have access to narratives of the past. This reached its zenith for some teens who had fantasies of rebirth:

The documents to start his life: *“I don’t believe that I’m in Paris now, for me it’s incredible; it’s a dream in fact; it’s as if I’m not yet born, all this. (F.)*

*“Compared with the interviews that I had before this study, there was a language barrier then. They asked me general questions about what happened in my life before, earlier. Here, the interviews, you’ve gone over my whole life. The future, I can’t tell it, because I don’t know what’s going to happen, but my childhood, how I grew up, all those aspects were considered. It’s allowed me to rewrite my history.”* (K).

The link between the past, present and future allowed him to construct his identity and to reactivate his psychological resources:

*K*: “Yes, it’s clear that if you want to be a farmer, you need to be strong. There, in my country, I was someone who was a big guy.

*Researcher: And you’re not anymore?*

*K: Yes, I’m still strong*!”

For those youth who mentioned psychological distress, the use of objects as part of the interview had a dual effect: of holding and of transformation. These objects thus helped the teens to jump-start the process of narrativity.

The objects chosen for the past were often memory objects, those of the present useful objects and those of the future something proving the youth’s membership in French society. For the past, music was the object mentioned most often, but each piece of music was related to these youths’ personal experience (“*what he says speaks to me, the songs about kids like me”*, in reference to Cheb Bilal, a singer himself exiled for 12 years without legal documents), a personal experience that is part of the country’s collective history (Crazy Soldier denounces the violence and the political situation of the country that M. had to leave to survive). Sometimes the object testifies to the trauma experienced and the migration (the dinar and the wound).

The objects of the present were most often utilitarian things associated with lodging, training programs, or learning French. These responses were less personal, but had a greater social valence. For the objects of the future, most mentioned obtaining a diploma, a “Young adult contract”, a residency permit, or French nationality.

For several youths, the choice of objects helped them to find a meaning to their fragmented pathway during which dreams were sometimes lost, replaced by discouragement. Accordingly K., who said that he had almost given up describing his route at the first interview, used as his object of the present at the second interview a drawing he made of himself with a beard to show that his departure far from his family served as a journey of initiation, a rite of passage. “*I started to shave, a deep shave. I have a little beard, hairs, to say that I’m older, so I can find a job.”*

Sometimes the choice of object helped in the coconstruction of a project shared with the social worker. Thus the young F., a young orphan from the Congo whose parents died in a traffic accident, said in her first interview that she is not motivated by her current schooling plan (providing personal services). During the second interview, she brought a fabric representing the 6 provinces of the Congo for the past, a choice linked to the cloth worn by her mother when she left on business. She then changed her plans and chose to study commerce. This cloth also allowed her to describe the distress and particular strengths of only daughters (her mother was also an only daughter) and to talk about her kinship line.

We also note that transcultural questions related to naming or food were especially strong generators of narrative.

#### From “I’ to “We”

##### Leaving the Sense of Loneliness Behind Between the First and Third Interviews

The discourse concerning the objects, the circle test, and the narrative developed in collaboration with the researchers and the social workers showed an evolution between the first and last meeting for the experience of isolation and loneliness.

Specifically, we note a greater prevalence of the use of “I” by these adolescents, to describe their distress, an “I” that is closed, trapped (“*I am inside”*); it evokes their loneliness (the social worker’s help seems disembodied). This “I” is organized around the “we”, with the collective and the peer groups in the old country, or around a “one” still more undifferentiated, in the present time, when they identify themselves with migrants or unaccompanied minor foreigners. They are becoming part of another peer group, not from their country of origin, but a mixed group of youth who have had similar pathways, at the same time that they were different. In the future, the “I” regularly becomes “one” when associated with a pathway that is organized in close association with their social worker. For some, this connection also takes place within larger social groups, that of friends from different training programs and for others around the other youths at the shelter they live in.

During the third interview, in many cases, a connection had clearly been established at the interpersonal level with the youth (in particular by the social worker). “*You helped me, all of you. When I come, I’m soothed, I sit down,” “before, I was taking rivotril (clonazepam) and now I don’t take it any more since I started with you*” (young Algerian, third interview). Another young Algerian was finally able to formulate a request for assistance to his social worker (the terms “*help,*” “*need,”* and “*one*” are repeated at the third interview).

**Figure 3:**
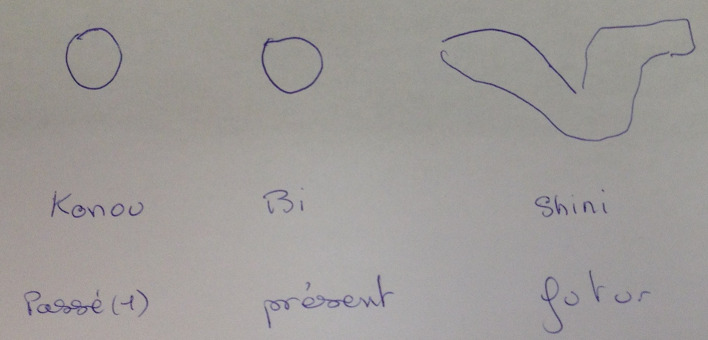
First circle test of a young malian (B.).

Several circle tests by themselves represented an evolution toward adoption of the values of the host country, probably addressed to the social worker and part of a process of cultural métissage. Let us illustrate this by the story of the young Malian, B., included in the study because he seemed to be isolated and to express a sadness and somatizations likely to affect his plan to learn to be a plumber. We learned from the mediator during this first circle test that this youth dreaded drawing a circle (**Figure 3**). The words for circle or *round* in Bambara and Soninké are “*korri,*” which is translated literally as “enclosure,” a closed space: closing someone in a circle or turning him around it is considered a bad omen. He therefore drew the future in the form of deployed wings.

**Figure 4:**
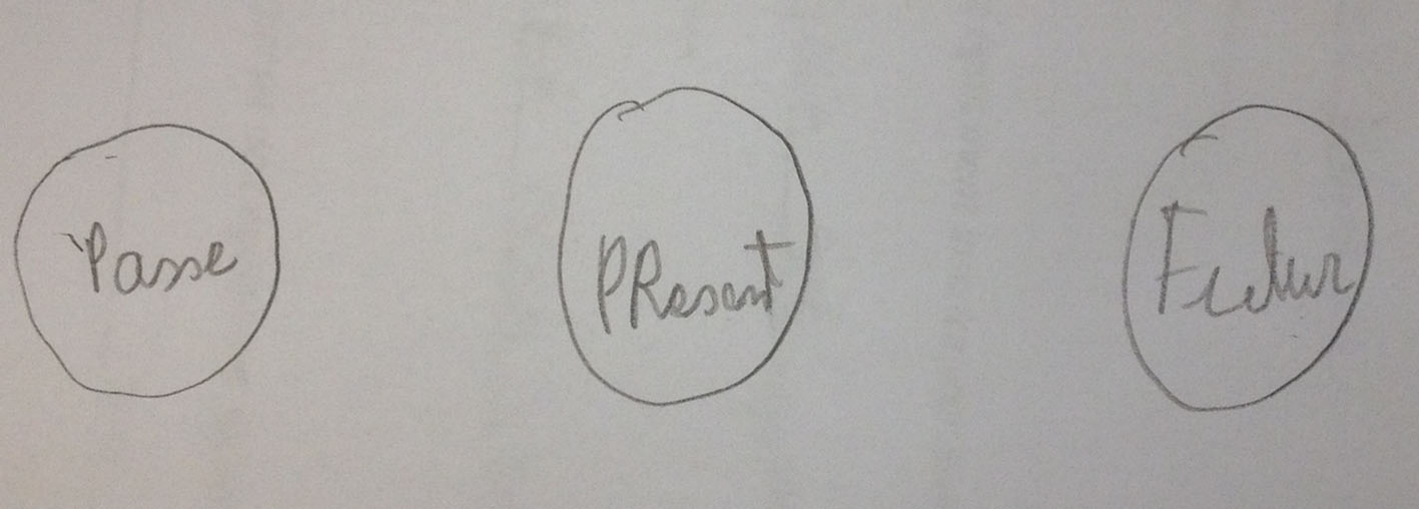
Second circle test (B.).

His circle test at the third interview was in the shape of three round, regular circles about the same size (**Figure 4**). He wanted to write the times in French in the circles. He thus expressed his ability to close them in the circle without fear that this would affect him later. We note greater confidence in his future, which is no longer vulnerable to bad omens or evil eyes, although they were previously likely to shut him in and prevent any progress. During this cycle of interviews, he succeeded in describing some aspects of his history. The social worker was able to describe in detail the difficulties in management that flowed from their professional positions and the youth’s shift in relation to the representations he had had of the concept of adolescence.

##### Talk About Oneself so That the Teens Can Talk About Their Own Cultural Representations in the Form of Exchanges, Conversation, and Sharing

This dialectic around the successful meeting might have occurred because the research process had enabled multidirectional mutual knowledge: between the teens and the social workers, between the social workers and the researchers-clinicians, and between the teens and the researchers-clinicians.

At the first interview, many of the social workers expressed their lack of knowledge of the youth, despite their intention to know him and to be engaged in his care.

“*I’m supposed to know him,” “I have the impression that I don’t know him.” (social worker of T. from Morocco and of Al., from Algeria)*

The youth were on the whole sensitive to the changes in their social workers. Some social workers had shared experiences from personal journeys or anecdotes with other youth. The young migrants looked at their social workers more as humans, now that these professionals were more exposed in relation to their position to otherness and their own experiences of decentering.^5^ Behind the professional is the human, and there is a cultural being who cannot from the outset know all of the youth’s cultural codes; and vice versa. Some social workers revealed themselves in some ways, while remaining professional: that is, they exposed themselves as professionals at the right dose, showing a little bit more of their identities, of their relation to the minors’ countries, their knowledge of the cultural universes to which they belonged, etc. This self-revelation was possible because it was initiated or encouraged by the researchers. This enabled the lifting of a cultural taboo: what one can allow oneself to say (or not). In truth, these youth are sensitive to others’ authentic interest in them, their culture, their cultural identities, and there is less self-censorship and more willingness to allow oneself to express one’s ignorance or doubt about the other’s culture. It involves learning from the other so that the other would like to learn from us.

Researcher: “*What do you get from working with this young man?*”

Social worker: “*It’s cultural enrichment. It’s true that Pakistan, I didn’t know it very well, or its history or its past, or any of that. So it’s true that it’s a cultural exchange that is very interesting. And then after, from a personal point of view, the choice of the profession of social worker, it’s also personal enrichment, that’s why you choose this profession, generally.*”

Mirroring this “humanization” of the social workers in the eyes of these young people is the personalization of the social work care and the humanization of the youth’s record:

K.’s social worker was able to underline his chaotic pathway and submit to the judge a solid case proving both that he was a minor and that his situation was very precarious. She was especially proud to have succeeded in humanizing his file to make the judge more aware: “*The fact that we showed K.’s pathway, everything that was done here at the Maison de Solenn, all that allowed us to humanize his file for the judge.”*

##### Acknowledge the Challenge of Integrating and Adapting to a New Society, a New School System, a New Social Environment With Unfamiliar Codes

We find the recurrent theme of the recognition of the youth’s suffering and the paradoxes of the system (subthemes of the relation to the policy world); this leads social workers to sometimes share the adolescents’ agitation and confusion, but without their impotence.

“*Arriving here, I discovered the reality. They told me a little that it was difficult down there, but I thought it was stories. But I’ve seen with my own eyes that it’s not stories, it’s reality, unfortunately*” (K., in his first interview, in which he described his voyage, during which he was treated badly across several European countries; nonetheless he reported that one of the greatest difficulties has been this precariousness at his final destination).

The recognition of the difficulties of the present was an essential stage and a prerequisite to developing a narrative that included the past. Those who did not need mental health care were better able to give a thoughtful character to their ordeals without being afraid of retraumatization by recounting it. This recognition also enables the initiation of a conversation on the relation to norms: in the present, related to what these teens want to take from their peers to construct their identity at the level of their affiliations but also what they think that adults in the host country expect of them and their fears of misunderstanding as they seek integration and respect. They must simultaneously consider not only who they are and who they want to become, but also what adults expect them to be. A particular fear is related to religion, which has been transmitted by their ancestors and is part of their filiation for the construction of their identity. The researchers regularly show that these two movements can be reconciled.

Social worker: “*In our association, it’s our principle to accept each person in their own culture, in their differences, with their past and their experience. Concretely, it’s respect for their religion.*”

For these youth, hearing that explicitly stated by someone who works in an institution of the Republic enables them to recount their affiliations, without self-censorship. It is not, on the other hand, mentioned in this way when interviews are conducted in a more formal, more administrative framework, on the subject of support. The young man here responded:

“*For a while I was at a hotel, I was allowed three meals a day and they gave me Halal meat. Another kid was in a different social work sector and his social worker said to him from the beginning: “ now, you are in France, you need to eat like the French do.*”

##### Assuming a Mother-Child Relationship

A maternal representation was quite regularly attributed to the social workers in this study. We might apply the concept of a good-enough mother, described by Winnicott^6^ in the mother-baby relationship. In these situations, it may involve a substitute mother able to meet these young people’s needs and support them in their progressive discovery of their new world. After a period of wandering in France, the social worker is their first stable reference, and it is by and through the social workers that these youth begin to discover the world in France in small doses. These institutional representatives/mediators thus provide a “presentation to the world” together with their framework for interpreting it to the youth; they support these young people in an environment that is culturally unknown and perhaps traumatizing in this setting of migration.

Accordingly one mediator reported a strong and emotional relationship between Al. and his social worker, but also the impression that he envisioned his relationship with her as unchangeable: *“He has a very strong relationship with her, at the same time as … it’s as if he were sure of keeping her with him.”*

They sometimes compared their social workers directly to a mother:

“*Like my mother because there are things she says to me, my mother also said to me” (unaccompanied youth from the Congo)*

“*He considers her like a mother and with all the respect and what goes with it.” (unaccompanied youth from Guinea).*

It should be noted that in some cultures, it is sometimes a mark of respect to designate a kind adult to whom one is close by a term designating the generation above (uncle, mother, aunt…).

This maternal identification might initially have caused surprise or discomfort to social workers, in view of the representation of the maternal bond in their own culture and the sensitive issue of emotions in their professional training:

“*I never realized where you were putting me in relation to you.*”

“*Here the word mother does not have at all the same meaning.*”

“*In our profession, it is considered a professional flaw to show affection for a child, for a youth.”*

For several of these adolescents, the need for a mother figure originated in a lack in their childhood family and in the need to take responsibility for themselves early on, without a family to support and shore them up in developing an ever more appropriate response to situations.

For several social workers, this feeling of discomfort evolved during the interviews and an emotional relationship with the adolescents did not become more threatening.

This was the case for the social worker of a young Cameroonian; she felt committed to a role that was not hers: “… *he put me in a very particular place, right away he said to me, I need you.”*

Later, she said: “*I’d like to say that the association took care of him; it carried him and showed him the world, perhaps as only a mother can show the world to her child.*”

But often it was the fact that the social workers were not the only professionals dealing with the child that made it possible for them to take on this emotional relationship. Here it was the association or voluntary agency that is treated as the parent. Sometimes a social worker mentioned that another social worker shared responsibility for the case. We can imagine that being the only person to “hold” the especially loaded history of an adolescent who is massively invested in a transference creates the risk of too great an investment by the social worker and too great a fascination, which is not neutral. In the study, things happened as if it required the gaze of a third party (the researcher) who has also heard the youth’s story for the social worker to take on this relationship with the youth and to know that it was possible without any role confusion. The agency offers itself as the “good-enough mother” where after the perfect adjustment sought by the social workers, they move toward progressive separation. The objective is to enable an exit from the state of dependence that these teens may have toward their social workers, without inducing unbearable anxieties for them, and thus avoid inflicting yet another of the ruptures they have experienced so many times along their journeys. The team can thus maintain a representation of the agency’s containment without its physical presence.

Sometimes, it is the research setting that evokes a reassuring familial ambience: *“he feels as if he’s at home, with family and he talks as if he’s with family”* (mediator).

#### A Mediator Who Facilitates Bridges Between Cultural Worlds

##### Social Workers Who Use Interpreters Rarely

The analyses of the self-administered questionnaires and of their experience of the study, as reported at the last interview, show that social workers are not used to using professional interpreters, even though they recognize that the language barrier is one of the principal difficulties. Language is one of the themes that appears most often in the transcripts. It is through language that we see the prism of the world. Nonetheless, the social workers only discovered during these research interviews the specific languages these adolescents had mastered.

“ *I met B. and we didn’t need an interpreter to talk because he spoke French, and I wasn’t curious about it; because I started and I didn’t know what it could refer to, ethnicity, the village, the origins; I said to myself that I had everything in the report…” (a social worker)*

The social workers used interpreters as mediators even less often, unaware of the value they provide in this role.

We can see this in the situation of T., a young Algerian who talked so much that his logorrhea left his social worker at a professional standstill (“*Because he talked to me so so much at each interview, he talks, I even have trouble stopping him so I can say something*”). Attributing communication problems in part to the language barrier, the social worker arranged for someone who could translate for the first interviews of the follow-up, but this effort was unsuccessful (“*I understood nothing at all, at all.”*). The function of a professional interpreter can go beyond the simple translation of words, which is already necessary for reasons of ethics and respect for the client (due to the risk that information will be lost or even deformed; there is also the problem of asking questions freely, through this filter). During this study, the teens easily grasped the value of the mediators, as the social workers observed. The mediator’s presence enabled them, for example, to manipulate the languages they spoke according to function and context. For example, they often reported their history and emotions in their native language, while stating concrete facts or words intended for the social workers in French.

##### A Role Model for the Youth

The questions about languages and cultural group of birth were among the first questions by the researchers and enhanced the meeting from the start:

‘‘*I found that right away, he was talking in Arabic, he was talking as if he’d met an old friend, or someone he already knew.*” (a social worker)

Mediators facilitate bridges with the country of origin. They are people who have succeeded in adapting themselves in several different universes. They are therefore adults who have succeeded their cultural métissage between here and there and who juggle between their two identities to turn them into creative wealth. They provide real help in negotiating between two cultural worlds. They are therefore figures the youth may identify with and aspire to be like. Beyond the language assistance they provide, the mediator ensures that these youths without families can dare to express their own points of view to an adult without being judged and be certain to be understood.

We also observed the importance of the continuity provided by using the same mediator for each interview.

##### Using the Mother Tongue Is Very Helpful for the Construction of a Multicultural Identity

Mediators help to avoid cultural misunderstandings. They are coprofessionals who take an active role in the interview and thus enable the researcher and social worker to listen more carefully. They can describe the habits and customs of the young person’s place of origin.

Interpreter: “*In fact, in the Punjab region of Pakistan, it’s not the same thing as in Punjab India; [in Pakistan], they don’t go to school much, they do the minimum. And afterwards, the children often take over their parents’ land to continue to perpetuate the tradition — farming.*”

Researcher: “*And what do they grow there?*”

Interpreter: “*It’s known for raising buffalo. In Pakistan, there are cows but most often we have buffalo. We use a lot of buffalo milk. This region is well known for that, for its milk.”*

The youth to the interpreter: “*that’s exactly right, but how do you know that?*”

Interpreter: “*I’ve worked in farming and I’ve raised buffalo too.*”

The migrant youth thus sees that the interpreter knows the world of France well, but also the world of Pakistan; the interpreter himself migrated from there. Interpreters can thus be very useful in the function of mediators: we ask the interpreter to translate word for word but also to facilitate the sharing of cultural representations and to allow a narrative to take shape. The interpreter can actively seek to create bridges with the country and can appreciate the youth and his or her skills. This often leads the teens to feel supported in expressing themselves. Furthermore, the interpreter-mediator enables the triangulation of the relationship between the social worker and the patient. This dual relationship is not natural in some countries. It is easier to express emotions in the mother tongue than in a second language.

Mediators thus appear essential in any interview with unaccompanied minor migrants or refugees, whether they know the only the rudiments of French or speak it fluently.

## Discussion

### Key Findings

In only three meetings, most of these situations were able to develop favorably, in the creation of a narrative, but also with the improvement of the relationship between young migrants and their social workers. The young migrants were led to unfold their story in a situation where it would not affect the definition of their status or their outcome. We did not force narratives in youths whose journeys had been traumatic, to avoid retraumatizing them, but the story emerged thanks to the organization of the framework described here. It is this framework as a whole that facilitates listening, understanding, and the youth’s speaking. The group also creates a containing environment for these migrant youth whose cultural envelopes appear torn. Moreover, in many of the traditional societies they come from, there is a permanent reference to the group, and issues are resolved by several group members.

The supports or prompts for narration (objects, circle test) adapted to this transcultural interaction proved to be very useful for these young people who did not express themselves easily. The young participants understood the instructions to bring an object with them. These methods therefore made it easier to gain access to and obtain a singular representation of their histories and pathways. It also facilitated the creation of a therapeutic alliance, the first stage of the beginning of long-term psychological or psychiatric treatment. These tools enabled the interactive creation and transformation of a self-narrative for each of these young migrants and most especially for those whose ability to narrate was impeded by various psychiatric disorders and mutilated by trauma. For those less affected by the trauma, the initial explanation for why social workers did not have access to these young people histories might lie in the cleavage of migration, a phenomenon described by Nathan (58): the experience of mental separation between before and after migration for every migrant. It resembles the loss of a psychological and sensory envelope with which one decodes the exterior world and stimulates the need to create another one, like a new skin. This experience of separation can be reactivated by each new period of separation (changing of group home or lodgings, for example). The associations made between past, present, and future in this study probably worked together to facilitate the construction of these young people’s identities. This takes place through the construction of an experience of the continuity of one’s existence [the *self-continuity* of Chandler and Lalonde (59)]. We constantly searched for coherence in their pathways so often characterized by family separations, cultural ruptures, and changes in their care. This coherence (in finding/refinding a direction in the fragmented journey) and this life story are necessary for the construction of their identity after the experience of trauma.

New clinical practices can thus be envisioned, as part of transcultural services. Moreover, within this program, we have been able to transmit to social workers the ways we work with interpreters-mediators, which they might possibly take up in their own institutions (12). Calling upon mediators in the framework of an institution appears to be an accessible means to improve care rapidly.

One of the important results of this study is that it served as a good introduction to mental health care: both for the teens who needed it but had not yet been identified; and for those who initially refused it because they were unable to represent its environment (for example, several with problems related to addiction or somatization). This study enabled us to define preventive or transition actions to provide them with better representations of mental health care and to introduce this care if appropriate when the time is right. The most appropriate timing to screen for posttraumatic stress disorder (PTSD) is still under debate (60). Stress reactions in the first 4 to 6 weeks after the traumatic event are considered a “normal reaction to abnormal events.” For psychotherapists, it is vital to start individual trauma therapy for young refugees only when it can be completed, which might be a challenge because of the insecure status of the length of their stay at a specific location.

Even though this research was not particularly addressed at a clinical population, these youth felt authorized to express their psychological distress in some situations and were able to ask for psychological care. The modality of this treatment was then explained by the mediators, and accurate representations by the youth (and the social workers) of its meaning facilitated the procedure. This research framework should make it easier to consider an analysis of the introduction of mental health care, its relevance, timing, and form. The transmission of transcultural skills to social workers can also, inversely, prevent unnecessary mental health care, especially through the use of mediation by objects and by their appropriation of this method of introducing transcultural jump-starts, through meetings such as those described in this study and conducted in a framework that facilitates the emergence of cultural representations. The introduction of an umpteenth professional (psychiatrist or psychologist in a clinical setting) for teens who have already met many (judges, police, group-home directors, social workers, group-home psychologists, etc.) can increase their confusion in identifying the roles of each, as well as adding to the number of ruptures in a pathway already strewn with them.

The question of the identification of those who do need psychological care (all do not need it despite possibly traumatic journeys) and how to introduce it is not obvious. For example, when they do need it, some young migrants refuse as part of a strategy of defense against trauma: not dealing with the past and denying anxiety becomes an adaptive coping strategy that enables them to limit their risk of collapsing into depression in the present: time splitting (61). Only a small percentage of refugees with psychological problems seeks for help (60).

Horlings and Hein (60) recommend short-term group interventions for young refugees suffering from PTSD. Group interventions have the advantage of being supportive when the problems are identifiable, recognizable, and the young people can be examples for each other. Moreover, they are less time-consuming and more cost-effective than individual treatments.

It is accordingly important to work on reassurance and the identification of the meaning of psychological treatment. Several studies have identified unaccompanied young migrants as a population at risk of developing psychiatric diseases and noted the importance of treating these disorders rapidly. Nonetheless, most of this population does not receive psychological care, and there is a dearth of studies assessing the effectiveness of treatment for them (62).

Major work on support at the sites the young migrants are housed can already be helpful; a first stage for a youth arriving in France is a place to settle and receive support to mitigate the risk of profound loneliness. It is often difficult for them to settle on arrival, with frequent and recurrent changes in housing and little time to discover a new world. We find the concept of trust central in the experience of these youth, who often describe a feeling of stigmatization and do not always feel that the various stakeholders listen to them, or even that they are allowed to talk about or practice their religion. Once their material survival is assured, depressive or posttraumatic symptoms that have been deferred may appear. The transition on their 18th birthday is another period of major psychological vulnerability, when some of these youth sometimes decompensate briefly into psychosis and require holding, in Winnicott’s sense of the word, to surmount this stage.

This follow-up cannot begin without the social worker, who is often the person to whom these youth turn first, as a resource person, in whom they confide, and who can therefore help them to formulate a request for psychiatric care and can support them in this framework. The social worker must thus be integrated into this management from beginning to end, for these young people whose parents are far away.

### Comparison With the Literature

There is a lack of research on screening and interventions in this specific population.

Hodge conducted a meta-analysis of 21 US studies assessing culturally sensitive interventions (CSI) for minority youth seen for violent behavior, substance use, and sometimes for physical medical problems (63). Its results suggest that currently operationalized CSIs are modestly effective with these youth. Although their results do not support the view that CSIs are more effective than standard treatments, these CSIs were tested as a first-line treatment for an entire population from a cultural minority. In our study, we suggest our intervention be used for specific indications rather than systematically. It is addressed to social workers who encounter problems in providing support services to unaccompanied foreign youth and who require specialist professional advice, as when there is a risk of a cultural misunderstanding or when the social worker has the impression that he or she does not know the youth or cannot succeed in creating a relationship of trust. Moreover, Hodge’s meta-analysis does not include any analysis according to either the youth’s degree of acculturation and/or generation of migration (first or second). In our study, the youths are unaccompanied first-generation foreigners who have recently arrived. These youths have therefore undergone a recent cultural shock, and these professionals must confront the otherness that accompanies them.

According to Horvat et al. (64), such cultural competence education programs need to be better specified and described. They should set forth including their conceptual rationale, actual content, delivery, organizational support and approach to evaluation. These authors examined five randomized controlled trials from different countries to assess the effects of cultural competence education interventions for health professionals on patient‐related outcomes, health professional outcomes, and healthcare organization outcomes. There was positive, albeit low‐quality evidence, showing improvements in the involvement of culturally and linguistically diverse patients. Findings showed either support for the educational interventions or no evidence of effect. They concluded that uncertainty exists about the best and most effective way to educate health professionals in cultural competence that leads to improved health outcomes for cultural minorities. Here, our study describes in detail a training program in cultural competence in which the social worker works directly with the youth, together with two experts in transcultural clinical practice. During this intervention, social workers learn on several levels: the nature of the relevant questions, the framework to modify, and how to work with an interpreter as a mediator. They will later be able to call upon these mediators more easily and will no longer be alone in transcultural situations. The result here is measured in terms of the improvement in the relationship with the youth and in terms of the therapeutic alliance, and not on clinical improvement, contrary to the studies identified by Horvat, which concerned quite different types of diseases, such as diabetes and hypercholesterolemia.

Successful interventions in young refugees do not require simply psychotherapeutic treatment focusing on PTSD symptoms. Instead to improve outcomes they should consist of individual as well as supportive factors, including but not limited to (reuniting) family, schooling, integration of traditional health care, and language training (65, 66). Our study underlines the value of some of the coping strategies described in the literature. Especially important is the need to maintain continuity despite an unstable and shifting situation (67, 68). Continuity can be facilitated by the maintenance of religious beliefs and practices in the host country (69, 70). Psychological functioning is improved by peer support; feeling safe at school, lack of discrimination, and low peer violence levels enable higher self-esteem (60). Accordingly, finding familiarity and regaining one’s bearings enable a transition that eases integration to the host country’s values and self-construction in a harmonious cultural métissage between two cultural worlds (71, 72). We also find in the literature the need for progressive acquisition of new cultural norms and the major role of contact with peers from their culture now integrated into the host country, who have often endured very similar situations and with whom they can share their experiences (67). It is often surprising, because we transform these youth into victims by underestimating their capacity of resilience. Most often, they talk little at the beginning, because they are sad and struggling. Telling them what we know of their collective history, by direct experience or texts and readings, can transform them (73). The very idea that they can be part of a collective history lifts their self-esteem. They understand that the professionals do not perceive them as wanderers but as people who seek to transform their destinies. We must make sure that they can restore their dignity and regain a desire to participate in a community by progressing past their distrust. We must not ask them to renounce their identity and their past, but rather we should help them to construct themselves with both. Interventions should start with a public health approach, focusing on basic security, environmental and supportive factors (60).

### Strengths and Limitations

This is the first study collecting the experience of 29 unaccompanied minors and providing them with detailed and original transcultural services adapted specifically to this population. A quantitative study might usefully complement this research by evaluating and assessing the impact of these services. Our idea is to adapt these services for prevention and when necessary, for treatment.

The analysis includes the eight youths who were lost to follow-up and had only a single interview because they are representative of situations of wandering. This wandering or rootlessness is often a part of the pathway of these unaccompanied minor migrants and is therefore part of our data and a result in itself. We have been able to analyze the first circle tests and what prevented a second interview. In most cases, the problems were related to the pathway through institutions and in three cases, to difficulties linked to their own errancy. Leconte explains the continuous wandering of some youth as a result of their need to pursue their route of exile to avoid encountering the moment of stability that will cause them to collapse psychologically (44).

### Conclusions/Implications for Practice

This transcultural approach appears to be a relevant framework for reactivating the process of identity construction. It promotes the emergence of cultural representations, while taking experiences before, during, and after migration into account. As a preventive process, this approach also makes it possible to limit routine use of mental health care and use it instead only when necessary and when it will be most effective.

The multiplicity of the problems concerning these unaccompanied minors lends itself to a qualitative methodology to explore these complex situations. This original study protocol is simultaneously useful for these adolescents, their social workers, and researchers. The migrant youth’s identity construction process is based on affiliation, belonging, and alternative affiliations (and the professional is the adult who might help to build a positive future). Professionals responsible for migrant young people must have an active part in the therapeutic process. In a transcultural approach, therefore, we emphasize their skills, we let them build their caring capacity, and we focus on plans, procedures, and a shared understanding of their future. This study has led to the emergence of requests for training. We have thus organized a seminar in Paris and drafted a handbook for social work professionals to encourage and enhance these meetings that improve the social workers’ knowledge of these adolescents and promote their successful interaction. The reception of these steps further underlines the importance of creating spaces for sharing thoughts, ideas, and interprofessional exchanges.

After the conclusion of this study, this service was resumed in the framework of the clinical and transcultural groups at the Maison de Solenn. It takes place in small groups, uses creativity and flexibility, is designed to facilitate narration, and takes special care for continuity, through the systematic presence of both the social workers and the mediator. These “transcultural jump-starts” are inspired by the transcultural group system, adaptable to different situations (74, 75) and based on the skills and uniqueness of the pathway of these children; they are intended to enhance resilience and promote the appreciation and showcasing of the heroic valence of a migration. Rather than uprooting the youth, we choose as in this study to help them in their cultural métissage and to construct new knowledge, based on their skills. Clinical work with these youngsters is often very rich. Inducing the invocation of their relatives’ existence in their narratives alleviates their feelings of isolation and fosters the sense of belonging to a group. We can thus assist them in their ability to produce an inclusive narrative of a bicultural adolescence and navigate smoothly between two worlds.

## Data Availability Statement

All datasets generated for this study are included in the article/**Supplementary Material**.

## Ethics Statement

The studies involving human participants were reviewed and approved by CEERB Paris North IRB00006477. Written informed consent to participate in this study was provided by the participants’ legal guardian/next of kin. Written informed consent was obtained from the individual(s)’ and minor(s)’ legal guardian/next of kin, for the publication of any potentially identifiable images or data included in this article.

## Author Contributions

All authors listed have made substantial, direct, and intellectual contribution to the work and approved it for publication.

## Funding

The study received financial support from the Fondation de France and the City of Paris.

## Conflict of Interest

The authors declare that the research was conducted in the absence of any commercial or financial relationships that could be construed as a potential conflict of interest.

## Footnotes

1 In France, 14,908 unaccompanied minors were reported in 2018, compared with 8,054 in 2016 (source: Ministry of Justice).

2 NAMIE Research Action: New Welcome for Unaccompanied Foreign Minors. Laboratory Inserm U 1178, Center Babel (S Bouznah), Hospital Cochin: Pr MR MORO (director of research), R. RADJACK (scientific supervisor), F. TOUHAMI, S MINASSIAN, L WOESTELANDT, C. LEBRUN, A. BERNICHI, S MALEY, A. MOSCOCO, S HIERON, F. HOLLANDE, J LACHAL, G LECONTE, T BAUBET, G STURM, A BENOIT, C LEDU, S ZIEMER, J MAILLARD.

3 Center Babel (European resource center in transcultural clinical care), Maison de Solenn (Cochin Hospital, Paris)

4 Professional interpreters trained in intercultural mediation.

5 Decentering is a process of flushing out the position that denies cultural otherness and sanding down or eroding human complexity by reducing from the unknown to the known. It's about leaving behind “cultural commonplaces.” Professionals must “decenter” themselves from their own representations to avoid interpreting through a single prism, which can lead to errors or overgeneralization. Experiences traveling or working in transcultural situations make it possible to recognize that there are many ways to think of social work and care or treatment, all of them valid in some ways or situations.

6 Winnicott D. La mère suffisamment bonne. Paris: Payot & Rivages; 2006
