# Supplementary material for: Cultural Competence of Professionals Working With Unaccompanied Minors: Addressing Empathy by a Shared Narrative
Source: Front Psychiatry. 2020 Jun 11;11:528. doi: 10.3389/fpsyt.2020.00528 (PMC7301836; doi:10.3389/fpsyt.2020.00528)
Supplement: Supplementary file 1 [file Table_1.docx]

**Table 3:** Choice of objects at the three interviews

| **Youth** | **Object of the past** | **Object of the present** | **Object of the future** |
| --- | --- | --- | --- |
| Those who did not receive psychological care | | | |
| 1 | Official booklet from childhood | Pastry | Current official booklet |
| 2 | Class picture from Guinea | Class picture from France | Photograph of him in front of his mirror |
| 3 | Religious song from childhood | Report card + basketball | Nothing |
| 4 | Fabric for a loincloth | Report card | White coat (doctor's) |
| 5 | Algerian flag | Badge of the group home | Photocopy of the application for residency permit |
| 6 | Food and music (Papa Wemba) | Idea of a take-off | Nursing school program  "Young adult contract" ^[[1]](#footnote-1)^ |
| 7 | Drawing of him at the age of 10, at home in Pakistan, in traditional clothing, smiling | Drawing of him in France in a polo shirt and with a beard, smiling | Drawing in which he is wearing a suit |
| 8 | Opening chapter of the Koran | The Koran | Destiny: "only God knows it" |
| 9 | A watch (given to him in his country but which wasn't useful to him until he learned to tell time in France) | A book of 50 Malian recipes, written in French | A cap symbolizing the headdress of a house |
| 10 | A Tunisian dinar that has not left his pocket despite the trials he experienced and his sea crossing | His "young adult contract" | A diploma and a job |
| 11 | A drawing of him in Pakistan | A bracelet purchased in Paris | A residency permit |
| 12 | Bracelet and chain sent by his mother | Family photo with him in a traditional turban | French nationality |
| 13 | Music: Crazy Soldier of Takanaza | His medication to treat his sickle-cell anemia, diagnosed in France | His medication |
| 14 | The Koran, a pair of glasses, a licorice stick | The same objects | The same objects |
| 15 | A loincloth fabric portraying the 6 provinces of the Congo | Identity card (with her name and those of her parents) | Plan to go into sales |
| Those who were referred for psychological care/treatment | | | |
| 16 | Music (the hopes of Coronthie) | Lemon and pen | Professional training in cooking |
| 17 | Song of Cheb Bilal related to his experience of exile and violence | (a different) song of Cheb Bilal | A diploma for "putting myself back together" |
| 18 | Cricket and school | Cake for Epiphany (language learning, a job, and belonging to a peer group at work) | A job in Paris |
| 19 | Photographs of good memories of Oran (house, mother, close friends)  Music by Cheb Hasni | Picture of his social worker and the hotel he sleeps in | A hole |
| 20 | Nebel, poor neighborhood in Douala | Words and singing | Vague plans |
| 21 | The psychological wound in his heart | His bag with his school things | Iron (related to his future work in maritime welding) |

1. The "Young adult contract" is a — rare — continuation of child welfare support past 18 years (renewable at most to the age of 21 years) [↑](#footnote-ref-1)
